# Recent Progress in the Development of Poly(lactic-*co*-glycolic acid)-Based Nanostructures for Cancer Imaging and Therapy

**DOI:** 10.3390/pharmaceutics11060280

**Published:** 2019-06-14

**Authors:** Ki-Taek Kim, Jae-Young Lee, Dae-Duk Kim, In-Soo Yoon, Hyun-Jong Cho

**Affiliations:** 1 College of Pharmacy and Natural Medicine Research Institute, Mokpo National University, Muan-gun, Jeonnam 58554, Korea; ktkim0628@mokpo.ac.kr; 2 College of Pharmacy, Chungnam National University, Daejeon 34134, Korea; jaeyoung@cnu.ac.kr; 3 College of Pharmacy and Research Institute of Pharmaceutical Sciences, Seoul National University, Seoul 08826, Korea; ddkim@snu.ac.kr; 4 Department of Manufacturing Pharmacy, College of Pharmacy, Pusan National University, Busan 46241, Korea; 5 College of Pharmacy, Kangwon National University, Chuncheon 24341, Korea

**Keywords:** cancer, diagnosis, nanoparticle, PLGA, targeting, therapy

## Abstract

Diverse nanosystems for use in cancer imaging and therapy have been designed and their clinical applications have been assessed. Among a variety of materials available to fabricate nanosystems, poly(lactic-*co*-glycolic acid) (PLGA) has been widely used due to its biocompatibility and biodegradability. In order to provide tumor-targeting and diagnostic properties, PLGA or PLGA nanoparticles (NPs) can be modified with other functional materials. Hydrophobic or hydrophilic therapeutic cargos can be placed in the internal space or adsorbed onto the surface of PLGA NPs. Protocols for the fabrication of PLGA-based NPs for cancer imaging and therapy are already well established. Moreover, the biocompatibility and biodegradability of PLGA may elevate its feasibility for clinical application in injection formulations. Size-controlled NP’s properties and ligand–receptor interactions may provide passive and active tumor-targeting abilities, respectively, after intravenous administration. Additionally, the introduction of several imaging modalities to PLGA-based NPs can enable drug delivery guided by in vivo imaging. Versatile platform technology of PLGA-based NPs can be applied to the delivery of small chemicals, peptides, proteins, and nucleic acids for use in cancer therapy. This review describes recent findings and insights into the development of tumor-targeted PLGA-based NPs for use of cancer imaging and therapy.

## 1. Introduction

There has been much progress in the development of nanomedicines for use cancer imaging and therapy [1,2,3,4,5,6,7,8,9,10,11,12,13,14,15,16,17,18,19,20]. To increase the potential for clinical application, nanomedicines with increased precision and safety have recently been designed and evaluated. Following intravenous administration, particles with a certain size range can localize to the tumor region through an “enhanced permeability and retention (EPR) effect” [21,22,23]. Molecules or particles with a size of >40 kDa (renal clearance threshold) may be present in the systemic circulation for a prolonged period [22,23]. Leaky tumor vasculature and insufficient lymphatic drainage of tumor tissue can increase the permeability and accumulation of particles [24]. Although the EPR effect has been proved in many animal studies, its usefulness for clinical application is still controversial [21]. The pathophysiological states are different according to the animal species (rodent versus human), tumor types originated from same source, and primary versus metastatic tumors in the same patient [21]. Consideration of the heterogeneity of EPR effect is necessary for the successful translation of nanomedicines to the clinical situation [21]. As drug delivery via EPR does not usually occur in normal tissues, it can be used as a passive tumor-targeting strategy [25]. However, the heterogeneity of tumor tissue, including highly hypovascular areas and necrotic tissues, frequently observed in large tumors may limit the efficiency of particle delivery. In addition to various factors that modulate the EFR effect [26] (e.g., bradykinin, nitric oxide derivatives, prostaglandins, angiotensin-converting enzyme inhibitors, and vascular endothelial growth factor [VEGF]), ligand–receptor interactions have been introduced as an active tumor-targeting strategy [27]. Ligands can be selected to bind receptors that are overexpressed in cancer cells compared with normal cells [27]. Receptors in tumor cells (e.g., transferrin [Tf] receptor, folate receptor, lectins, and epidermal growth factor receptor [EGFR]) or on the tumoral endothelium (e.g., VEGF receptors, α_v_β_3_ integrin, vascular cell adhesion molecule-1 [VCAM-1] and matrix metalloproteinases [MMPs]) can be targets of ligand-tethered nanosystems [27]. Recently, internal (e.g., pH, enzyme, and redox state) and external (e.g., temperature, magnetism, and ultrasound) stimuli-sensitive smart nanosystems have been designed to provide more sophisticated drug-release patterns and selective uptake in cancer cells [27]. Additive properties, such as tumor penetration, the induction of apoptosis, and inhibition of metastasis, have been built in nanosystems for use in cancer imaging and therapy [28,29,30,31,32,33,34,35,36,37,38].

To reduce toxicity in the development of tumor-targeted nanomedicines, biocompatibility and biodegradability are considered principal issues in the selection of materials for nanosystems. Various types of synthetic polymers (e.g., poly(lactic-*co*-glycolic acid) [PLGA]), natural polymers (e.g., chitosan [CS], chondroitin sulfate [CD], and hyaluronic acid [HA]), lipids (e.g., phospholipid and cholesterol), nucleic acids (e.g., DNA), peptides/proteins (e.g., albumin and lysozyme), and inorganic materials (e.g., gold, iron, silver, and zinc) have been used to prepare nanoformulations for cancer therapy [39,40,41,42,43,44,45,46,47,48,49,50,51,52,53,54,55,56,57,58]. Among these diverse materials, PLGA is a favored substance for the fabrication of nanoparticles (NPs) aimed at drug delivery [59,60,61]. PLGA can be degraded into lactic acid (LA) and glycolic acid (GA), which can enter metabolic pathways. Therefore, it can be safely applied to the preparation of injection formulations. PLGA has received approval from the United States Food and Drug Administration and the European Medicine Agency for its application in injectable formulations [62]. It can be chemically modified to give biofunctionality (i.e., tumor-targeting capability), and the outer surface of PLGA-based NPs can be transformed to provide a prolonged circulation time and tumor targetability [63,64,65,66]. Small chemicals, peptides, proteins, and nucleic acids with diverse physicochemical properties can be entrapped in PLGA or PLGA derivative-based NPs or adsorbed onto the outer surface of NPs [61,67,68,69,70]. Convenient modification and reliability in the fabrication of NPs, as well as their favorable biosafety, may elevate the feasibility of applying PLGA-based NPs in the clinic.

Several articles have reviewed the application of PLGA nanomedicines to drug delivery [59,61,62,67]. Herein, we introduce recent progress in the development and evaluation of PLGA-based NPs for cancer imaging and therapy. Information regarding the preparation, cellular uptake and distribution, in vivo tumor targeting, in vivo cancer therapy, and in vivo pharmacokinetics of PLGA-based NPs will be provided.

## 2. Preparation and Modification of PLGA-Based NPs

### 2.1. Preparation of PLGA NPs

The fabrication method of PLGA NPs may affect their physicochemical properties, such as particle size, particle size distribution, particle shape, drug encapsulation efficiency, and particle stability [71]. The most common methods for the preparation of PLGA NPs, such as emulsification, nanoprecipitation, spray-drying, and microfluidics, are described in following sections.

#### 2.1.1. Emulsification-Evaporation Method

Emulsification is the most frequently used method. This method can be subdivided based on the emulsion (single; e.g., oil in water [O/W] emulsion and double or multiple; e.g., water in oil in water [W/O/W] emulsion) and acquisition of the NPs (i.e., solvent evaporation and dialysis). PLGA polymers and hydrophobic drugs can be dissolved in water-immiscible organic solvents, such as chloroform or dichloromethane, followed by addition to the aqueous phase containing a surfactant such as poly(vinyl alcohol) (PVA) to obtain an O/W emulsion. This emulsification can be prepared using a homogenizer or ultrasonic probe, which can provide high shear energy. Then, the organic solvent can be evaporated with simple stirring, under a gentle nitrogen gas stream, or in a vacuum state. The hardened NPs can be obtained by centrifugation, washed to remove remaining surfactants, and lyophilized for further storage. The organic solvent in the oil phase of emulsion systems can also be diffused by dialysis against a large amount of water. Solidified particles containing PLGA polymer and drugs may be precipitated and acquired [60,62].

The double or multiple emulsion method can be used to encapsulate further hydrophilic drugs. An aqueous solution containing hydrophilic drugs is added to the PLGA-dissolved organic phase under vigorous stirring to obtain a W/O emulsion. This emulsion is added to the second aqueous solution containing a surfactant under high-shear energy to form a W/O/W emulsion. Then, the hardened NPs can be obtained by solvent evaporation [72].

The emulsification method can be used to encapsulate both hydrophobic and hydrophilic drugs. Furthermore, it can be used to prepare particles, varying from nano to micron size by adjusting the stirring speed and input rate [71]. Nonetheless, there are some problems with this method, such as the requirement for heat or a vacuum for solvent evaporation and the imperfect elimination of stabilizer [72]. Recently, membrane extrusion has been used to prepare NPs with a narrow size distribution after the emulsification process. Membrane extrusion is performed using a polycarbonate membrane under high pressure [60].

#### 2.1.2. Nanoprecipitation Method

PLGA and hydrophobic drugs can be dissolved in a water-miscible solvent such as dimethyl sulfoxide (DMSO), acetone, and ethanol. This organic solution is usually injected into an aqueous solution containing surfactant at a controlled speed with an auto-injector. NPs can be formed by the rapid diffusion of polymers and drugs out of organic phase. Then, they can be obtained by solvent elimination [73].

Nanoprecipitation is used to load hydrophobic drugs into the NPs via a one-step procedure. This method can be useful to prepare PLGA NPs with a <100 nm particle size, which can be determined by the polymer concentration [60]. The advantages of this method include its easy scale-up, low energy requirement, high reproducibility, and single step process. The limitations include the low encapsulation efficiency of the hydrophilic drug and particle size variation, which is dependent on the mixing process [72].

#### 2.1.3. Spray-Drying Method

The spray-drying method is a rapid process that can be used to encapsulate both hydrophobic and hydrophilic drugs. Aqueous solution containing hydrophilic drugs may be added to the PLGA-dissolved organic phase under vigorous stirring to obtain the W/O emulsion, and can be used to make multiple emulsion. Particles are obtained by spraying W/O emulsion or multiple emulsion with a stream of heated air [74]. However, the main limitation of this method is the adhesion of particles to the walls and cyclone part of the spray dryer, resulting in low productivity [68].

#### 2.1.4. Microfluidics Method

The continuous-phase flow microfluidic system, including a flow-focusing system and co-flowing system, can be used to prepare nano-sized PLGA particles by forming single emulsions. In the flow-focusing system, the dispersed phase flows through a narrow capillary and the continuous phase flows from the two side channels with the vertical direction of the dispersed phase. In the co-flowing system, the dispersed phase flows inside the capillary and the continuous phase flows outside the capillary in the same direction [60].

The microfluidic method has several advantages for preparing PLGA NPs, such as a narrow size distribution, high reproducibility, and the prevention of an initial burst release. Moreover, the microfluidic system can be used to prepare various surface-modified PLGA NPs, such as PEGylated NPs and NPs carrying targeting moieties [72]. However, it also has limitations, including limited production scale (and scale-up), and problems associated with microchannel clogging and fouling [72].

### 2.2. Surface Modification of PLGA NPs for Tumor Targeting

#### 2.2.1. Physicochemical and Biological Properties of PLGA

PLGA is a copolymer composed of LA and GA and those two units are linked via an ester bond. Therefore, PLGA can be degraded into LA and GA by hydrolysis in the body and can join in metabolic pathways [67,75]. The degradation of PLGA NPs may be influenced by the molecular weight (MW), molar ratio between LA and GA, crystalline degree, glass transition temperature, and the type of end cap (acid versus ester) [62,67]. Poly(lactic acid) (PLA) is more hydrophobic than poly(glycolic acid) (PGA); thus, a PLGA copolymer with a higher ratio of LA/GA may be degraded more slowly [62]. Additionally, PLGA with an ester end cap may have higher resistance to hydrolysis compared with PLGA with an acid end cap [62]. This biocompatibility and biodegradability can increase the feasibility of using PLGA NPs in clinical applications. By modulating the polymerization process, various types of PLGA in terms of MW, LA:GA ratio, end cap, and chirality, can be prepared. Those properties can affect polymer degradation, drug release from NPs, cellular uptake, and the in vivo fate of NPs [59,60,61,62,65,66,67,68,69]. Furthermore, several block or grafted copolymer types (e.g., linear, branched, and star-shaped) based on PLGA can be synthesized with CS, HA, poly(caprolactone) (PCL), poly(ethylene glycol) (PEG), polyethylenimine (PEI), poly-l-lysine (PLL), poly(styrene), and PVA [59,62,76,77,78]. Amphiphilic copolymers (e.g., PEG-PLGA and PLGA-PEG-PLGA) can form a micellar structure following dispersal in an aqueous environment, and present different particle characteristics [79,80]. In addition, reactive units (e.g., amine, maleimide, *N*-hydroxysuccinimide, and vinyl) can be attached to the terminal end of a PLGA copolymer. These functional groups can participate in further modifications with tumor-targeting ligands or drug cargos. These structural changes can modulate the physicochemical and biological properties of PLGA-based NPs [62,67,69].

#### 2.2.2. Surface Engineering of PLGA-Based NPs

For active tumor-targeting strategies (i.e., ligand–receptor interaction) together with a passive tumor-targeting approach (i.e., EPR effect), the outer surface of PLGA-based NPs has been engineered with various functional groups. Two methods can be used to prepare surface-engineered PLGA NPs; 1) After synthesizing PLGA derivatives (linked to functional groups), they can then be used to fabricate NPs. 2) After fabricating PLGA NPs, the exposed functional groups (i.e., carboxylic acid and hydroxyl groups) of PLGA are chemically linked to targeting moieties, or the outer surface of PLGA NPs are coated with functional materials.

For prolonged circulation in the blood, hydrophilic molecules such as PEG, poloxamers, and Tween 80 are usually conjugated on the surface of PLGA NPs. These hydrophilic molecules on the NPs can infer stealth properties, leading to the evasion of phagocytic uptake by the reticuloendothelial system (RES), and a prolonged time in the systemic circulation [81]. Furthermore, PLGA NPs conjugated or coated with positively-charged molecules such as CS can be taken into the tumor cells more easily via adsorptive-mediated transcytosis [82].

Active targeting for tumor-specific delivery can be achieved by introducing targeting moieties to the surface of NPs, which are specifically recognized by receptors on the tumor cells or tumor vasculature, resulting in the increased penetration of tumor cells via receptor-mediated endocytosis [83]. The receptors or surface-bound antigens may be expressed exclusively or overexpressed on the tumor cells compared with normal cells. Various targeting moieties such as folic acid (FA), Tf, biotin, HA, peptides, aptamers, and antibodies (Abs) have been used on the surface of PLGA NPs to actively target tumor microenvironments (Table 1). However, the expression of receptors on the tumor cell or vasculature can be altered and alternative receptors can be upregulated [72]. Recently, NPs carrying multiple (e.g., dual or triple) ligands have been investigated to maximize tumor-targeting efficiency.

**Figure 1:**
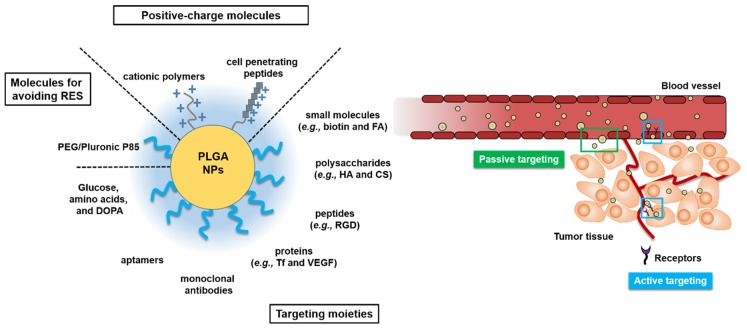
Various surface-engineered poly(lactic-*co*-glycolic acid) (PLGA) nanoparticles (NPs) for passive or active tumor targeting. Arg-Gly-Asp (RGD); chitosan (CS); dopamine (DOPA); folic acid (FA); hyaluronic acid (HA); poly(ethylene glycol) (PEG); reticuloendothelial system (RES); transferrin (Tf); vascular endothelial growth factor (VEGF).

The development of multi-functional PLGA NPs modified with cell-penetration peptides, Pluronic^®^ P85 (an inhibitor for drug efflux pump), or PEG are promising strategies for use in cancer therapy [84]. These PLGA NPs can passively target delivery to tumors via the EPR effect, and actively target delivery to tumors based on ligand–receptor interactions, and overcome drug resistance with inhibitors of efflux pumps, finally improving anti-tumor efficacies and reducing systemic toxicity in normal tissues and organs [85,86]. Surface-engineered PLGA NPs for passive and active tumor targeting are illustrated and summarized in Figure 1 and Table 1, respectively.

## 3. Uptake of PLGA NPs into Cancer Cells

**Figure 2:**
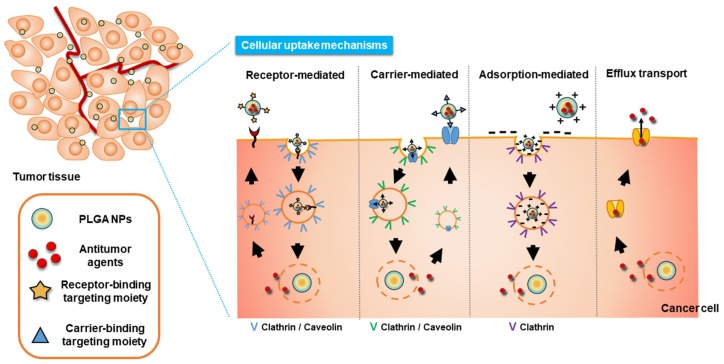
Cellular uptake mechanisms of surface-modified PLGA NPs.

The uptake of surface-engineered PLGA NPs into tumor cells can be described through several endocytosis mechanisms, such as receptor-, carrier-, and adsorption-mediated endocytosis (Figure 2). Increased cellular uptake of NPs can be observed using those endocytosis mechanisms, and thereby regarded as tumor-specific drug delivery strategies through passive or active targeting. Particle size, particle shape, the surface charge of particles, hydrophilicity/hydrophobicity of particles, elasticity of particles, and cell type can affect and determine the endocytosis pathway and efficiency [46,97,98,99,100,101]. Particles with 10–100 nm are reported to be necessary for entering the endocytic vesicles and smaller particles are known to be beneficial for their rapid cellular entry [100]. In addition, polydispersity and surface charge of particles may affect the cellular entry [100].

### 3.1. Receptor-Mediated Endocytosis

Most NPs carrying anti-tumor agents, including PLGA NPs, can be internalized by endocytosis into the tumor cells. In receptor-mediated endocytosis, the NPs can be efficiently internalized into cells by the specific binding of targeting moieties anchored on the PLGA NPs and the corresponding receptors expressed on the tumor cell membrane or tumor vasculatures [102]. These specific ligand–receptor complexes include the biotin–biotin receptor, HA-CD44 receptor, anti-epidermal growth factor (EGF) antibody (Ab)-EGF receptor (EGFR), FA-folate receptor, Arg-Gly-Asp (RGD)-based peptide-integrin α_v_β_3_, and Tf–Tf receptor (Table 1). 

Clathrin and caveolin are endogenous molecules that exist in the cytosol and function in receptor-mediated endocytosis. When NPs conjugated to targeting moieties interact with the receptor, each clathrin or caveolin molecule migrates from the cytosol to the cell membrane, encompasses the ligand–receptor complexes, and contributes to the production of clathrin-coated vesicles or caveosomes containing the complexes [97,98]. Clathrin-dependent endocytosis is a representative route of entry into mammalian cells [100]. In clathrin-dependent endocytosis, coated clathrin may be peeled off prior to fusion with early endosomes and NP will reach lysosomes via the endo-lysosomal route [97]. Conversely, in caveolin-dependent endocytosis, NPs can be internalized as a form of caveosome, which can avoid fusion with lysosomes and prevent lysosomal degradation [97,103].

### 3.2. Carrier-Mediated Endocytosis

Some hydrophilic molecules, such as amino acids, peptides, monoamines, and glucose, which can be used as building blocks, neurotransmitters, and energy sources for cells are usually recognized selectively and transported actively via interaction with carrier proteins expressed on the cell membrane [104]. Tumor cells require high levels of energy and building blocks for their abnormal rapid growth, and thereby overexpress those carrier proteins, including hexose carriers and amino acid carriers. Thus, glucose can be used as a promising targeting moiety for the tumor-specific delivery of PLGA NPs. Glucose-included PLGA NPs can be specifically bound to glucose transporters such as GLUT-1 and GLUT-3 or sodium-glucose linked transporter (SGLT) -1 and -2 on certain tumor cells, followed by internalization into cells via carrier-mediated endocytosis [70].

For the treatment of brain tumors such as glioblastoma, therapeutic molecules should cross the blood-to-brain barrier (BBB), which is the most impermeable barrier in the human body [105]. Fortunately, for the purpose of transporting neurotransmitters (i.e., choline and l-dopa) and energy sources (i.e., glucose) into the brain, specific carriers, including choline transporter, l-system large neutral amino acid transporter, GLUT, and SGLT are present on the BBB [70,106]. Therefore, NPs loaded with anti-tumor agents for brain tumor-specific delivery could be modified with targeting moieties, which can be bound to those carriers on the BBB and receptors (i.e., CD44 receptor, folate receptor, and Tf receptor) on the tumor cells [27,51,89,91].

### 3.3. Adsorption-Mediated Endocytosis

As the surface of cell membranes at physiological pH carries a slight negative charge, electrostatic interactions between cationic NPs and tumor cell membranes can promote particle adsorption to the tumor cells [107]. Non-functionalized PLGA NPs have a negative surface charge due to the structure of PLGA polymer, resulting in lower penetration into the tumor cells due to the electrostatic repulsion. Therefore, NPs functionalized with a cationic polymer such as CS and cell-penetrating peptides (CPPs) (e.g., low-molecular-weight protamine [LMWP], transactivator of transcription [TAT], and penetratin) can easily penetrate tumor cells via adsorption-mediated endocytosis [90,95,108]. Although there are some exceptions, positively charged NPs may be dominantly internalized via a clathrin-mediated pathway [100]. For example, CS-coated PLGA NPs were found to be internalized via both macropinocytosis and clathrin-mediated endocytosis, while the uptake PLGA NPs involved caveolin-mediated endocytosis in MDCK cells [108]. The positively charged surface of NPs may be associated with some issues, such as reduced circulation time due to the enhanced opsonization and recognition by the RES. Recent progress regarding particle properties-dependent endocytosis types is summarized in Table 2.

## 4. Visualization of Tumor Targeting by In Vivo Imaging

### 4.1. Design of NPs with Imaging Modalities

**Figure 3:**
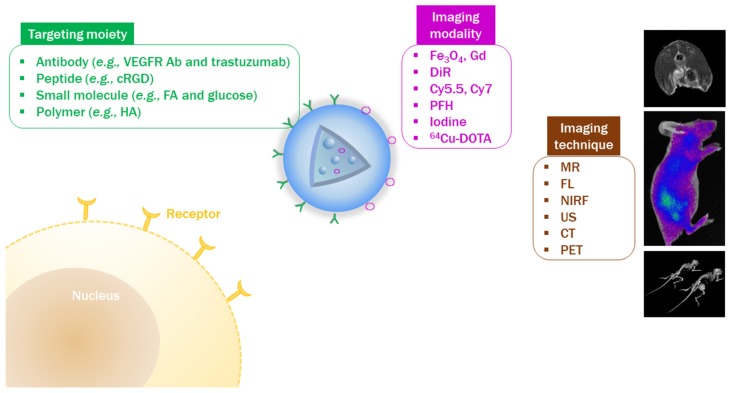
Evaluation of tumor-targeting efficiency by in vivo imaging techniques. Antibody (Ab); computed tomography (CT); cyclic Arg-Gly-Asp (cRGD); fluorescence (FL); folic acid (FA); gadolinium (Gd); hyaluronic acid (HA); magnetic resonance (MR); near-infrared fluorescence (NIRF); perfluorohexane (PFH); positron emission tomography (PET); 1,4,7,10-tetraazacyclododecane-*N*,*N’*,*N’’*,*N’’’*-tetraacetic acid (DOTA); ultrasound (US); vascular endothelial growth factor receptor (VEGFR).

The tumor targeting ability of PLGA-based NPs can be demonstrated using in vivo imaging techniques. To monitor the in vivo fate of NPs, a corresponding imaging modality should be introduced to NPs (Figure 3). Imaging agents can be attached to the PLGA backbone (prior to NP fabrication) or to the outer surface of NPs (after NP fabrication) [59,115]. As commercial PLGA products possess a carboxylic acid or ester end group, they can be used to generate covalent bonding with the functional groups of imaging modalities [59]. For instance, amine or hydroxyl groups in the imaging agent can directly form an amide or ester linkage with the terminal carboxylic acid group of PLGA [99,116]. The imaging modality can be also attached to the functional groups (e.g., amine group) of PLGA derivatives or appropriate linkers (e.g., bifunctional PEG linker) can be used to connect the imaging modality and PLGA [65]. In addition, the outer surface of PLGA NPs can be modified with imaging agents [115]. Exposed functional groups of PLGA NPs can participate in the reaction with imaging modalities [117,118]. Covalent bonding between PLGA (or PLGA NPs) and an imaging agent can reduce the loss of imaging agent prior to the localization of NPs to the tumor region following injection. To acquire a sufficient resolution for in vivo imaging, it is important to optimize the degree of substitution by the imaging agent. Imaging agents can be physically introduced to PLGA-based NPs [115]. After PLGA NPs are fabricated, the outer surface of NPs can be coated with the imaging agent by adsorption. However, this may risk an initial burst of imaging agent release following injection into the blood stream, and reduce the efficiency of visualizing target sites. Together with the solubilization of PLGA in the organic phase, several imaging agents can be added to the organic solvents (e.g., acetone, chloroform, dichloromethane, DMSO, and ethanol) during the fabrication of NPs [33,119,120]. To encapsulate an imaging modality in PLGA NPs, drug encapsulation efficiency and release rate also should be evaluated prior to application for in vivo imaging.

For magnetic resonance (MR) imaging, *T*_1_-or *T*_2_-weighed contrast agents can be introduced to PLGA NPs. In general, gadolinium (Gd) derivatives or iron oxide (Fe_3_O_4_) NPs have been loaded to NPs to enhance the contrast in MR imaging [120,121,122]. 1,1′-Dioctadecyl-3,3,3′,3′-tetramethylindotricarbocyanineiodide (DIR) has been widely used for in vivo fluorescence imaging [123,124]. Recently, many attempts have been made using near-infrared fluorescence (NIRF) imaging during the development of nanoformulations due to its deeper tissue penetration and lower tissue scattering property than fluorescence imaging [125]. In NIRF imaging, several kinds of fluorescence molecules (e.g., Cy5.5, Cy7, protoporphyrin IX [PpIX], and indocyanine green [ICG]) can be introduced to PLGA NPs [33,126,127]. Derivatives of NIRF dyes can be covalently conjugated to PLGA or encapsulated in PLGA NPs. For ultrasound (US) diagnosis, bubble-generating agents (e.g., perfluorohexane [PFH]) can be incorporated into the PLGA NPs [128,129]. In computed tomography (CT) imaging, several contrast agents (e.g., iodine derivatives and gold nanostructures) can be entrapped in PLGA NPs or coated on the outer surface of PLGA NPs [115,130]. Prior to application in in vivo imaging, the appropriate dose for visualizing the tumor tissue should be determined. Additionally, chemical modification and physical loading of imaging modalities should not affect the intrinsic biofunctions of PLGA NPs, including drug release, cellular binding, and in vitro/in vivo anticancer activities, in cancer theranostics.

Detection of signals from imaging modality-contained PLGA-based NPs in the body can indicate their biodistribution. Unfortunately, advantages and disadvantages for cancer diagnosis have been reported for each imaging modality [115]. For instance, MR imaging has high spatial resolution and relatively low sensitivity. In contrast, positron emission tomography (PET) imaging exhibits high sensitivity and low spatial resolution. Therefore, the combination of PET/MR imaging can provide a high sensitivity and high spatial resolution in the diagnosis of diseases. Therefore, in vivo imaging methods with dual or triple modalities have been combined to exploit the benefits of each imaging technique [128,131]. Multiple imaging modalities can enable more accurate tracing of NPs and the diagnostic ability of the disease state following systemic administration.

### 4.2. Introduction of Tumor-Targeting Ligands to PLGA NPs

To increase the tumor-targeting efficiency by active tumor-targeting strategies, diverse kinds of ligands can be introduced to PLGA-based NPs (Figure 3). Together with the “EPR effect” as a passive tumor targeting strategy, ligand–receptor interactions can improve tumor-targeting efficiency in cancer imaging and therapy [25,27]. Small molecules, polymers, proteins, peptides, and Abs can be linked to the PLGA backbone or the functional groups of PLGA derivatives [132]. Amine-, aldehyde-, carboxyl-, maleimide-, succinimidyl ester-, or sulfhydryl-functionalized PEG-linked PLGA can be coupled with various kinds of tumor-targeting ligands [132]. Amine-functionalized folate-polyethylene glycol (PEG) was conjugated to PLGA-PEG-COOH via a carbodiimide reaction to synthesize a folate-coupled PLGA-PEG derivative [133]. The amine group of an antibody (e.g., Herceptin^®^) can be linked to the carboxylic acid groups on the outer surface of PLGA derivative-based NPs via *N*-(3-dimethylaminopropyl)-*N’*-ethylcarbodiimide hydrochloride (EDC) and *N*-hydroxysuccinimide (NHS)-coupled reactions [129]. For peptide labeling, cyclic Arg-Gly-Asp (cRGD)-thiol (SH) was reacted with maleimide-functionalized PLGA-PEG NPs to prepare cRGD-linked PLGA-PEG NPs [128].

### 4.3. Verification of Tumor Targetability by In Vivo Imaging

Passive and active tumor-targeting approaches guiding drug delivery and cancer imaging can be assessed using several in vivo imaging techniques. In vivo and ex vivo imaging data can provide data on the pattern of biodistribution of fabricated NPs in the body. Interestingly, in vivo imaging can show the real-time distribution of fabricated NPs and the current status of cancer tissue without the need to sacrifice experimental animals. PLGA-based NPs guided by real-time imaging can increase the therapeutic efficacies of drug cargos. Recently, several studies have reported the in vivo tumor targeting capability of PLGA-based NPs by bioimaging techniques (Table 3).

Lipid-platinum prodrug (Pt(IV)) conjugate-loaded PLGA-PEG-cRGD NPs have been designed, and glutathione (GSH)-sensitive and US-triggered anticancer activities have been assessed [128]. cRGD-modified Pt(IV) NPs can target the tumor site via an EPR effect (passive tumor targeting) and cRGD-integrin receptor interactions (active tumor targeting). Under US exposure in the tumor region, the NP shell can be cracked, releasing the lipid-Pt(IV) prodrug. This can then adsorb onto the cellular membrane due to its amphiphilicity and move into the internal cellular space via passive diffusion. cRGD-modified Pt(IV) NPs were transported into the internal space of cancer cells via receptor-mediated endocytosis. Prodrug and NPs were released from the endosome in response to endogenous GSH. A combination of US and cRGD-modified Pt(IV) NPs enhanced the production of reactive oxygen species (ROS), resulting in mitochondrial-mediated apoptosis in cancer cells. The tumor-targeting efficiency of cRGD-modified Pt(IV) NPs was further verified with Cy7 incorporation by NIRF imaging in SKOV-3 tumor-bearing mouse models.

HER2-targeting PLGA NPs including PFH, paclitaxel (PTX), and superparamagnetic iron oxide (SPIO) were fabricated and their photothermal/chemo-therapeutic efficacies guided by photoacoustic (PA)/US imaging were evaluated [129]. Herceptin was conjugated to the outer surface of PLGA-based NPs to target the HER2 receptor. SPIO and PFH were included in the NPs for PA and US imaging, respectively. SPIO can convert external near-infrared (NIR) light to thermal energy and can thus be used for the photothermal ablation of tumors. It can then be combined with the chemotherapeutic effects of PTX for the treatment of breast cancers. Photothermal heating of SPIO can be transferred to PLGA polymer, which can make the structure of the polymer rubbery. Then, a drug with poor aqueous solubility (PTX) can be diffused from the NPs. The tumor-targeting efficiency of Herceptin-modified NPs was assessed by photoacoustic (PA)/US bimodal imaging in the SKBR-3 tumor-xenograft mouse model [129]. Under NIR laser irradiation, Herceptin-modified NPs containing PHF, PTX, and SPIO exerted superior tumor growth inhibition based on the synergism of photothermal- and chemo-therapeutic efficacy.

Angiopep-2-linked PLGA@Au NPs including docetaxel (DTX) were prepared and then NIR laser-triggered drug release and chemo-photothermal therapeutic efficacies were evaluated [130]. Organic-inorganic hybrid NPs based on a core-shell structure were designed with an outer Au shell that can be used for photothermal therapy (PTT) by converting the NIR laser to heat energy and as an X-ray imaging agent. Angiopep-2 carried by NPs can be efficiently internalized in U87MG cells via receptor-mediated endocytosis and have presented improved anticancer activity. X-ray and NIR thermal imaging analyses were performed to demonstrate the tumor-targeting efficiency and therapeutic potentials. Angiopep-2-conjugated Au nanoshell/PLGA/DTX NPs exhibited the anti-glioma activity in U87MG tumor-bearing mouse models [130].

PLGA NPs coated with amphiphilic HA derivative (HA-ceramide [HACE]) were developed to target the CD44 receptor for drug delivery [65]. HACE was composed of a hydrophilic backbone (HA) and a hydrophobic segment (CE); thus, PLGA NPs can be entrapped in the internal hydrophobic cavity of self-assembled HACE nanostructures. DTX was encapsulated in PLGA-based NPs as a poorly water-soluble chemotherapeutic agent. Higher levels of HACE-coated PLGA NPs were internalized by cancer cells expressing the CD44 receptor- (MDA-MB-231 cells) compared with cancer cells negative for the CD44 receptor (NIH3T3 cells). In mice bearing MDA-MB-231 tumors, HACE-PLGA NPs accumulated at higher levels in tumor tissue compared with PLGA NPs.

## 5. In Vivo Anticancer Activities of PLGA-based NPs

### 5.1. Parameters for Assessing In Vivo Antitumor Activity

Various PLGA NPs developed as anticancer therapies have been applied to animals to demonstrate superiority versus conventional interventions. Currently, standard techniques used to assess in vivo antitumor activity include tumor size measurement and survival analysis. Specifically, cancer cell-lines are xenografted in animals, who are often immunocompromised, and the tumor volume or survival rate is monitored over time in response to treatment. Tumor volume is determined using the modified ellipsoid formula: (tumor length × tumor width^2^)/2 [138,139]. However, in some models where direct tumor measurements are not available (e.g., tumor inside the skull, lung, or liver), the tumor in the scanned image or the weight of the dissected tumor can be used to substitute for volume data [140,141]. The survival rate is defined as the percentage of survivors in a group over a specific period after diagnosis. From the survival rate versus time function (i.e., Kaplan–Meier curve), the median survival value can be estimated and used to assess the effectiveness of the treatments [142,143]. 

### 5.2. Key Factors for Improved Antitumor Efficacy

**Figure 4:**
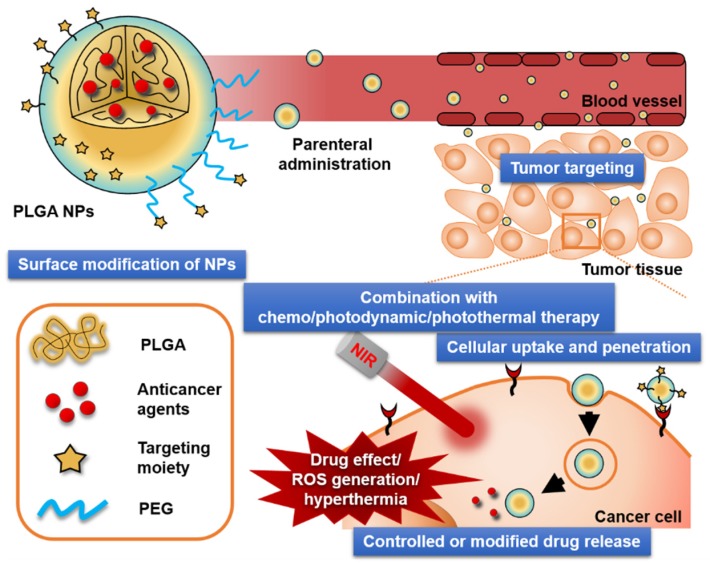
Crucial factors that affect the antitumor activities of PLGA NPs. Polyethylene glycol (PEG); reactive oxygen species (ROS).

Most PLGA NPs developed for anticancer therapy share therapeutic advantages, such as passive targeting via the EPR effect and controlled drug release (Figure 4). Moreover, further chemical or physical modifications with other compounds provide specific functions not provided by the unmodified NPs. These properties have been translated into significant improvements in antitumor activity, as shown in Table 4.

#### 5.2.1. Controlled Release and EPR Effect

The clinical value of anticancer agents is often highly compromised due to the dose-limiting severe side effects (e.g., renal and gastrointestinal toxicities). Many approaches using PLGA NPs have been developed to avoid the accumulation of drug in normal tissues (Table 4). Moreno et al. reported that cisplatin (CP)-loaded PLGA NPs exhibited comparable antitumor efficacy to free CP [144]. However, the PLGA NPs presented reduced systemic toxicity in terms of body weight and blood urea nitrogen (BUN). The authors suggested that the results were due to the controlled release of CP and the passive targeting of PLGA NPs, as well as the induction of apoptosis. 

PEGylation is one of the most extensively studied strategies to enhance the antitumor efficacy of PLGA NPs, and increases the circulation time of NPs in the bloodstream. This pharmacokinetic advantage increases the chance of tumors being targeted by NPs. Interestingly, CP-loaded NPs prepared with the mPEG-PLGA copolymer resulted in an enhanced survival rate compared with free CP, which was not observed when using the unmodified PLGA NPs [144,145]. Similarly, mPEG-PLGA NPs co-loaded with gemcitabine (GEM) and betulinic acid (BA) resulted in a significant reduction in tumor volume compared with GEM + BA solution in mice with Ehrlich ascites carcinoma xenografts [146].

#### 5.2.2. Targeted Delivery

The surface modification of PLGA NPs with tumor-targeting moieties (i.e., active-targeting strategy) can also enhance anticancer activity [147]. Typical examples can be found in studies using PTX-loaded PLGA NPs, in which the targeting moieties were tethered with the aid of PEG linkers. Liang et al. reported that the addition of FA, a small molecule ligand of folate receptor, to the surface of PTX-loaded PLGA NPs resulted in increased reduction in tumor volume compared with unmodified NPs in HEC-1A tumor-xenografted mice [148]. A comparable improvement was observed in studies exploiting peptides (iNGR; CRNGRGPDC) as a tumor-targeting moiety. The median survival of mice bearing intracranial U87-MG glioblastoma was 1.6-fold longer following treatment with PTX-loaded iNGR-PEG-PLGA NPs compared with unmodified NPs [149]. Aptamer (AS1411) conjugation also augmented the tumor inhibitory activity of PLGA NPs. The average tumor volume in C6 tumor-xenografted mice following treatment with PTX-loaded AS1411-PEG-PLGA NPs was reduced by 33% compared with those treated with the non-targeted NPs [140].

The targeting moieties can be attached indirectly. The most straightforward approach was based on the charge-charge interaction between the targeting moiety and the NP surface. This was demonstrated by Wu et al., who coated the surface of PLGA NPs modified with a positively charged surfactant, vitamin E-oligo(methyl diglycol l-glutamate) with the anionic polymer, HA [143]. In that study, the HA-coated PLGA (HPLGA) NPs loaded with DTX prolonged median survival 1.4-fold compared with free DTX in orthotopic A549-Luc lung xenograft model. Another approach has exploited the strong binding affinity between avidin and biotin. Biotin-conjugated RVG29 (fragment of the rabies virus coat protein) was successfully attached to avidin-palmitate anchored on the surface of camptothecin (CPT)-loaded PLGA NPs; however, no significant difference was found in median survival compared with the biotin-attached CPT-loaded PLGA NPs (control) in mice bearing intracranial glioblastoma [142]. However, doxorubicin (DOX)-loaded lecithin hybridized PLGA NPs resulted in a reduced mean tumor area (9.6 ± 10.7 mm^2^) compared with free DOX (21.7 ± 13.4 mm^2^) in orthotopic glioblastoma rat models [141]. This was attributed to the adsorption of apolipoprotein A-1, an endogenous targeting moiety in the bloodstream, to the lecithin-rich surface of NP after systemic administration.

#### 5.2.3. Cellular Uptake and Penetration

Factors regarding cellular uptake and penetration are also closely related to the antitumor activity of PLGA NPs. One interesting approach was reported by Zhang et al. [150], who co-loaded methenamine mandelate (MM), a urinary antibacterial agent, with NaHCO_3_ in PLGA NPs. Following the entry of NPs cancer cells via the endo-lysosomal pathway, NaHCO_3_ generates CO_2_ bubbles due to the acidic nature of endo-lysosomes. The bubbles broke down the structural integrity of the NPs and the subsequent release of MM resulted in formaldehyde production in the cancer cells. This pH-responsive drug release resulted in greater inhibition of tumor growth in MCF-7 tumor xenografted mice compared with NPs without NaHCO_3_. Another approach reported by Wang et al. utilized CPP to decorate the surface of PLGA NPs [90]. In that study, tumor penetration and intranuclear delivery of DOX were achieved by the alkyl-chained LMWP anchored in PLGA NPs, resulting in near complete arrest of tumor growth in MCF-7/ADR tumor-xenografted mice.

#### 5.2.4. Combination with Photodynamic Therapy (PDT) and/or PTT

PDT and PTT have gained much attention based on their effectiveness in cancer treatment [7,43,151,152]. Combination treatment with chemotherapy, PDT, and PTT using multifunctional PLGA NPs has been investigated. A photosensitizer (e.g., 5-aminolevulinic acid, hypocrellin A, meso-tetra hydroxyphenylchlorine [mTHPC], pheophorbide a [Pba], and PpIX) and/or photothermal agents (e.g., ICG) can be introduced into PLGA NPs by encapsulation or adsorption [153,154,155,156,157,158]. FA-modified PLGA NPs including Pba have been fabricated, and enhanced anticancer activities based on PDT against gastric cancer (MKN28 tumor) after intravenous injection have been demonstrated [155]. Peng et al. reported the use of polydopamine (PDA) and d-α-tocopheryl polyethylene glycol 1000 succinate (TPGS)-coated DTX-loaded PLGA NPs [159]. When co-treated with NIR laser irradiation, this nanoplatform resulted in an approximately 10-fold reduction in tumor size and weight compared with Taxotere^®^, a commercial formulation of DTX, in MCF-7/ADR tumor-xenografted mice. This result was explained by the augmented photothermal conservation property by PDA and the reduced multi-drug resistance by TPGS. Similarly, gold- or polyaniline-modified PLGA NPs also resulted in effective tumor suppression in the presence of NIR irradiation [130,160]. These therapeutic advancements were ascribed to the synergistic effects of chemo/photodynamic/photothermal therapy.

## 6. In Vivo Pharmacokinetics of PLGA-Based NPs

The pharmacokinetics of DTX-loaded PEGylated PLGA NPs (DTX-PEG-PLGA NPs) was assessed in Balb/C mice bearing C26 tumors [161]. The PEGylated NPs exhibited a markedly delayed plasma clearance (CL) as shown in Table 5. Following the administration of DTX-PEG-PLGA NPs, the plasma DTX levels remained much higher after 24 h compared with those of free DTX solution and DTX-loaded PLGA NPs. In contrast, the DTX solution and DTX-loaded PLGA NPs were quickly eliminated from the systemic circulation and their plasma concentrations 2 h after dosing were low. These data suggest that the NPs with a steric PEG barrier would prevent their rapid uptake by a mononuclear phagocyte system and improve their circulatory half-life. Shah et al. reported the pharmacokinetics of PTX-loaded PLGA NPs surface-modified with Tf and Pluronic^®^ P85 in male Sprague-Dawley (SD) rats bearing C6 glioma [89]. PTX in solution administered intravenously was rapidly cleared from blood whereas PTX loaded in different NP formulations was retained in blood for longer, indicating the long circulation properties of drug-loaded NPs (Table 5). This increase in residence time may be attributed to decreased opsonization from blood due to the smaller size (less than 200 nm) and hydrophilicity (imparting stealthiness) of the NPs [162].

Milane et al. reported the biodistribution and pharmacokinetics of polymer-blend (PLGA-PEG-EGFR-targeting peptide) NPs loaded with lonidamine in an orthotopic animal model of multidrug-resistant breast cancer [163]. As shown in Table 5, the NPs containing EGFR-targeting peptide (targeted NPs) increase the plasma AUC, MRT, and t_1/2_ of lonidamine relative to lonidamine administered as a solution. Furthermore, the targeted NPs decrease the tumor λ_z_, increase the tumor MRT, and increase the AUC relative to drug solution. Collectively, although exact mechanisms remain unclear, the NP formulations improve the tumor pharmacokinetics relative to drug solution, with the targeted NPs providing a slight advantage in terms of the pharmacokinetic parameters of lonidamine relative to the non-targeted NPs.

The comparative pharmacokinetics of platinum (Pt)-loaded PLGA-PEG NPs and Pt prodrug was evaluated in male SD rats following intravenous administration of the maximal tolerated doses of the compounds (40 and 20 mg/kg, respectively) [164]. Pt remaining in the systemic circulation 1 h after administration was 77% following administration of Pt-PLGA-b-PEG-NPs and 15.6% following administrated of the Pt prodrug, whereas this value for CP has been reported as 1.5% in the literature [165]. The mean AUC for total Pt in the blood was about 26% of that observed in plasma (Table 5), suggesting that Pt is not extensively distributed in red blood cells. After a single intravenous dose of the Pt-loaded NPs, both the liver and spleen showed the highest Pt concentration at 24 h. In the kidney, which is the primary target organ of cisplatin toxicity, the Pt-loaded NPs resulted in lower Pt levels than the Pt prodrug. The lower Pt level in the kidney indicated that the NP system would exhibit reduced nephrotoxicity compared with that of CP. Notably, the 24-h cumulative Pt excretion after Pt-loaded PLGA-PEG NPs was about 17 times less than after cisplatin treatment. This difference is probably due to the characteristics of Pt compounds, which can bind to protein or other tissue compositions firmly through covalent bonds.

Ma et al. reported the tissue distribution of ICG-loaded PLGA NPs modified with PEG and FA in mice xenografted with MDA-MB-231 tumors, which present high expression of folate receptor [166]. As shown in Table 5, the plasma AUC_0–12 h_ in the dual-modified PLGA NPs was higher than the corresponding AUC_0–12 h_ in the non-modified PLGA NPs, except for the AUC_0–12 h_ in liver, which was lower. Such an increase and decrease confirmed the function of PEG, which enabled the NPs to circulate longer in blood and partially escape elimination by the liver. The tumor AUC_0–12 h_ of the dual-modified PLGA NPs was three-fold higher than that of the non-modified PLGA NPs, strongly demonstrating the tumor-targeting ability of the dual-modified PLGA NPs. Since both PEG and FA were present on the dual-modified NPs, it is difficult for this study to reveal which factor contributed more for tumor targeting. However, it is speculated that longer systemic circulation is a prerequisite for tumor targeting via an EPR effect, and simultaneously FA enhances the affinity of the NPs with folate receptor-expressing tumor cells, resulting in the accumulation and intracellular uptake.

A pharmacokinetic study investigating bufalin-loaded NPs comprised of mPEG, PLGA, PLL, and cRGD was performed in mice with SW620 colon cancer [167]. In plasma, bufalin was detectable even 24 h after injection in mice treated with bufalin-loaded mPEG-PLGA-PLL-cRGD NPs (BNPs), while bufalin was not detected in the plasma of mice treated with free bufalin. The MRT and elimination half-life of BNPs were significantly greater than those of free bufalin (Table 5). Huang et al. reported the pharmacokinetics of biodegradable DTX-loaded self-assembled NPs made of PLGA/HA block copolymers (PLGA/HA NPs) in SD rats [168]; both DTX-loaded PLGA and the PLGA/HA NPs resulted in some increase in the plasma levels of DTX compared with free DTX. The V_d_ for the PLGA/HA NPs was only half of that reported for PLGA NPs or free DTX, indicating greater retention of active DTX in plasma circulation when loaded into PLGA/HA NPs. As shown in Table 5, the AUC for the PLGA/HA NPs was 2.5- and 3.8-fold higher than that of the PLGA NPs and free DTX, probably due to HA shell. Collectively, the PLGA/HA NPs could prolong the circulation of DTX in the plasma.

Chen et al. reported the pharmacokinetics of DTX-loaded PLGA-PEG NPs modified with anti-prostate-specific membrane antigen (PSMA) aptamer in LNCaP tumor-xenografted BALB/c mice for targeted delivery of DTX to prostate tumors [169]. The CL and V_d_ of DTX decreased when DTX was applied in encapsulated formulations, suggesting favorable conditions for enhanced systemic exposure, which is reflected by the increases in AUC (Table 5). The DTX solution presented higher distribution to the heart and lower distribution to other organs, including the liver, spleen, lung, and kidney, compared with PLGA-PEG-apt NPs. Generally, NPs administered intravenously are readily entrapped in the RES, a unique microstructure abundant in the spleen, liver, and lung [170].

In a previous study [171], curcumin, a clinically promising anticancer phytochemical, exhibited a very short half-life of ~8 min following intravenous dosing as a free form in female BALB/c nude mice. However, PLGA-PEG NPs conjugated with EGFR-targeting GE11 peptide for breast cancer therapy prolonged the half-life to ~6 h. This provided further evidence that the sustained release of curcumin from the NP formulation and/or protection from elimination in the systemic circulation could prolong the period of tumor exposure to curcumin. Recently, it was reported that anti-EGFR protein-anchored PLGA-PEG NPs (immunonanoparticle, INP) for breast cancer therapy significantly enhanced tumor PTX concentrations by approximately 93-fold, compared to PLGA-PEG NPs and free PTX in athymic mice [94]. Paolini et al. developed galactosamine-modified PLGA NPs with a hydrodynamic diameter of 63 nm for the liver-specific delivery of bergamottin, a natural CYP3A4-inhibiting compound [172]. The bergamottin-loaded PLGA-galactosamine NPs efficiently inhibited CYP3A4 in vitro, and the highest accumulation was observed in hepatocytes. Compared with DTX treatment alone in nude mice bearing MDA-MB-231 tumors, co-treatment with NPs significantly improved the delay in tumor growth and demonstrated a major improvement in overall survival (survival rate of 67% versus 0% at day 55). Ahmad et al. assessed in situ intestinal transport and in vivo pharmacokinetics of DTX-loaded CS-coated PLGA NPs (DTX-CS-PLGA NPs), which were developed for target therapy of intestinal cancer [173]. DTX-CS-PLGA and DTX-PLGA NPs, along with GF120918, presented a 5- and 2.2-fold enhancement, respectively, in apparent intestinal permeability in a rat ileum permeation study. Similarly, significantly higher C_max_, T_max_, and AUC were observed following administration of DTX-CS-PLGA and DTX-PLGA NPs compared with free DTX suspension in rats. The higher T_max_ is consistent with the sustained in vitro release profile, whereas the higher C_max_ and AUC for the nanoformulations may be attributed to the enhanced intestinal absorption of DTX. The possible mechanisms of the enhanced DTX absorption include encapsulation of DTX, protecting it from GI shielding, avoiding CYP and P-glycoprotein recognition, and endocytosis into enterocytes (direct uptake of NPs) [173].

## 7. Current Status and Challenges of PLGA-Based Formulations for Clinical Applications

There have been many developments in PLGA-based formulations for the diagnosis and therapy of diseases in research status [59,60,61,62]. Several pharmaceutical products based on PLGA have been prescribed as follows: Arestin^®^ (minocycline HCl, microsphere, periodontal; OraPharma), Bydureon^®^ (exenatide, microsphere, subcutaneous; AstraZeneca PLC), Eligard^®^ (leuprolide acetate, in situ forming gel, subcutaneous; Tolmar Pharmaceuticals, Inc.), Lupron depot^®^ (leuprolide acetate, microsphere, intramuscular; AbbVie Inc.), Ozurdex^®^ (dexamethasone, implant, intravitreal; Allergan), Signifor LAR^®^ (pasireotide pamoate, microsphere, intramuscular; Norvatis Pharmaceuticals Corp.), Vivitrol^®^ (Naltrexone, microsphere, intramuscular; Alkermes, Inc.), and Zoladex^®^ (goserelin acetate, implant, subcutaneous; AstraZeneca PLC). Micellar structure based on mPEG-poly(d,l-lactide) including PTX has been also developed and used in clinics as Genexol^®^ PM (Samyang Biopharm). Unfortunately, there was little progress in the commercialization of PLGA-based NPs for cancer imaging and therapy. For the clinical translation of developed PLGA NPs, following drawbacks should be overcome; poor drug loading capacity (in spite of high drug encapsulation efficiency), high initial burst release rate of drug, generation of acidic products after its biodegradation, difficulty in scale-up process, and nanotoxicology [174]. In view of the toxicological issue, the influences of degraded products (LA and GA) of PLGA to the major organs and tissues were investigated [175]. In that literature [175], the oral administration of PLGA NPs in rats exhibited minimal toxicities on liver and intestine and no toxicity in brain, kidney, and lung. Recent endeavors in the areas of regulation and mass production may accelerate the launch of PLGA-based nanoformulations for cancer therapy in the market.

## 8. Conclusions

A variety of nanosystems based on PLGA or PLGA derivatives have been developed for cancer imaging and treatment. Because of the biocompatibility and biodegradability of PLGA, its potential for clinical application may be high compared with that of other materials. As naive PLGA-based NPs can only target tumors passively through the EPR effect, several functional groups (i.e., targeting ligands) have been introduced as active tumor-targeting strategies. Chemical or physical modulation of PLGA NPs can control drug cargo release, endocytosis in cancer cells, in vivo tumor targeting efficiency, in vivo anticancer activities, and in vivo pharmacokinetics following their intravenous administration. However, for clinical application, mass production process of those PLGA-based NPs should be established and the safety of surface-engineered PLGA NPs has to be thoroughly identified. The introduction of biosafe or commercially available functional materials into PLGA-based NPs may augment their anticancer efficacy and attenuate unwanted adverse effects.

## Figures and Tables

**Table 1:** Surface modification methods of PLGA NPs for tumor targeting.

Formulation	Anticancer Agent	Target (Cell Line)	Surface Modification Method	Ref.
mPEG-PLGA NPs	Mitramycin	Pancreatic carcinoma (BxPC-3 and MIA Paca-2 cells)	Mitramycin was loaded onto mPEG-PLGA NPs by the single-emulsion solvent evaporation method using poloxamer 188 as a stabilizer	[87]
CS-modified PLGA NPs	PTX	Breast cancer (MDA-MB- 231 cells)	PTX-loaded PLGA NPs were prepared by the nanoprecipitation method using a high-gravity rotating packed bed. Then, PLGA NPs were modified with CS through electrostatic adherence	[88]
Pluronic^®^ P85 or Tf-modified PLGA NPs	PTX	Glioma (C6 cells)	PTX-loaded PLGA NPs were prepared by the nanoprecipitation method. PLGA NPs were coated with Pluronic^®^ P85 or conjugated with Tf	[89]
C_24_-LMWP peptide-modified PLGA NPs	DOX	Drug-resistant lung cancer (A549/T cells) and drug-resistant breast cancer (MCF-7/ADR cells)	Desalted DOX-loaded PLGA NPs were prepared by the nanoprecipitation method. Then, a C_24_-LMWP hybrid peptide was introduced to PLGA NPs by electrostatic interaction	[90]
HA-PEG- PLGA or CD-PEG-PLGA NPs	pDNA lipoplex	Glioblastoma (U87 cells)	HA or CD was conjugated to PLGA-PEG-NH_2_ using a reducing agent and a catalyst. HA-PEG-PLGA or CD-PEG-PLGA NPs were prepared by the dialysis method	[91]
PLGA-PEG-biotin NPs	SN-38 (active metabolite of irinotecan)	Breast cancer (4T1 cells)	NHS-Biotin and H_2_N-PEG-NH_2_ were conjugated. Then, PEG-biotin was conjugated to PLGA-NHS to synthesize PLGA-PEG-biotin. PLGA-PEG-biotin NPs were prepared by the modified emulsification solvent evaporation method	[92]
PLGA-PLL-PEG-Tf NPs	DNR	Leukemia (K562 cells)	DNR-loaded PLGA-PLL-PEG NPs were prepared by the modified double-emulsion solvent evaporation/diffusion method. Tf was conjugated to the surface of NPs with CDI	[93]
Anti-EGFR mAb-PLGA-PEG NPs	PTX	Triple-negative breast cancer (MDA-MB-468 cells)	PTX-loaded PLGA-PEG NPs were prepared by the nanoprecipitation method. The anti-EGFR mAb was anchored on the surface of NPs by crosslinking with MBS	[94]
CS-RGD-modified PLGA NPs	PTX or CDDP	Lung cancer (H1299 and A549 cells)	Drug-loaded PLGA NPs were prepared by the emulsification solvent evaporation (PTX) or double emulsion (CDDP) method. CS-RGD was synthesized by conjugating the GRGDSP peptide to chitosan via maleimide-PEG-NHS. CS-RGD was physically adsorbed onto the PLGA NPs	[95]
Glucose-PLGA	DTX	Human laryngeal carcinoma (Hep-2 cells)	DTX-loaded glucose-PLGA NPs were prepared by the single-emulsion solvent evaporation method.	[70]
CD133 aptamer- PEG-PLGA	Salinomycin	CD133-positive osteosarcoma (Saos-2 cells) and cancer stem cells	Salinomycin was loaded into PLGA-PEG-COOH NPs by the single-emulsion solvent evaporation method. CD133 aptamers were conjugated to PLGA-PEG-COOH NPs by EDC/NHS coupling.	[96]

Arg-Gly-Asp (RGD); chitosan (CS); chondroitin sulfate (CD); *cis*-diamine platinum (CDDP); daunorubicin (DNR); dicyclohexylcarbodiimide (DCC); docetaxel (DTX); doxorubicin (DOX); epidermal growth factor receptor (EGFR); 1-Ethyl-3-(3-dimethylaminopropyl) carbodiimide (EDC); hyaluronic acid (HA); low-molecular-weight protamine (LMWP); methoxypolyethylene glycol (mPEG); *m*-maleimidobenzoyl-*N*-hydroxysuccinimide (MBS); monoclonal antibody (mAb); *N*-hydroxysuccinimide (NHS); *N,N*-carbonyldiimidazole (CDI); paclitaxel (PTX); polyethylene glycol (PEG); poly(lactic-*co*-glycolic acid) (PLGA); poly-l-lysine (PLL); transferrin (Tf).

**Table 2:** Type of endocytosis depending on the particle properties of PLGA-based NPs.

Surface Functionalities	Mean Diameter (nm)	Type of Endocytosis	Ref.
AS1411 aptamer	128	Receptor (nucleotin)-mediated endocytosis	[109]
CD44 antibodies	140	Receptor (CD44)-mediated endocytosis	[110]
l-carnitine	189	Carrier (OCTN2)-mediated endocytosis	[111]
Glutamate- polyoxyethylene stearate	152–181	Carrier (LAT1)-mediated endocytosis	[112]
CS	140–173	Adsorption-mediated endocytosis	[88]
PEG-HIV-TAT	97–176	Adsorption-mediated endocytosis	[113]
Arginine-rich peptide	156	Adsorption-mediated endocytosis	[114]

Organic cation/carnitine transporter 2 (OCTN2); large amino acid transporter 1 (LAT1); chitosan (CS); polyethylene glycol (PEG); human immunodeficiency virus (HIV); transactivator of transcription (TAT).

**Table 3:** Verification of tumor-targeting capability by in vivo imaging.

Polymer	Drug	Targeting Material	Target Receptor (Cell Line)	Imaging Technique	Imaging Modality	Ref.
PLGA-mPEG	Platinum(II) prodrug	cRGD	Integrin (SKOV-3 cells)	US and NIRF	PFH and Cy7	[128]
PLGA-PEG-COOH	PTX	Herceptin	HER2 receptor (SKBR-3 cells)	PA and US	PFH and SPIO	[129]
PLGA-PEG-COOH	DOX	AS1411 aptamer	Nucleolin receptor (C26 cells)	MR	SPIO	[134]
PLGA-PEG	DOX	Biotin	Biotin receptor (4T1 cells)	FL	DOX	[135]
PLGA-PEG-COOH	DOX	FA	Folate receptor (Bel-7402 cells)	US and MR	PFH and Fe_3_O_4_	[133]
mPEG-PLGA-PLL	PTX	Anti-CA19-9 Ab	CA19-9 (capan-1 cells)	FL	DiR	[123]
Maleimide-PEG-PLGA	Curcumin	c(RGDf(N-me)V)	α_v_β_3_, α_v_β_5_, and α_5_β_1_ integrins (C6 cells)	FL	DiR	[124]
PLGA	DTX	Angiopep-2	LRP-1 (U87-MG cells)	NIRF and X-ray	IR780 and gold nanoshell	[130]
PLGA-Glc	DTX	Glucose	Glucose transporter (Hep-2 cells)	NIRF	Cy5.5	[70]
PLGA	DTX	HA	CD44 receptor (MDA-MB-231 cells)	NIRF	Cy5.5	[65]
PLGA-PEG	Curcumin and PTX	T7 (HAIYPRH) peptide	Tf receptor (U87 cells)	FL, X-ray, and MR	DiR and MNP	[136]
PLGA	ZnPc	Anti-VEGFR-2 Ab	VEGFR-2 (MDA-MB-231 cells)	PA and US	ZnPc and PFH	[137]

Antibody (Ab); cyclic Arg-Gly-Asp (cRGD); 1,1‘-dioctadecyl-3,3,3′,3‘-tetramethylindotricarbocyanineiodide (DIR); docetaxel (DTX); doxorubicin (DOX); fluorescence (FL); folic acid (FA); hyaluronic acid (HA); low-density lipoprotein receptor-related protein-1 (LRP-1); magnetic nanoparticle (MNP); magnetic resonance (MR); methoxypolyethylene glycol (mPEG); near-infrared fluorescence (NIRF); paclitaxel (PTX); perfluorohexane (PFH); photoacoustic (PA); phthalocyanine zinc (ZnPc); poly((d,l)lactic-glycolic)acid–star glucose (PLGA-Glc); polyethylene glycol (PEG); poly-l-lysine (PLL); superparamagnetic iron oxide (SPIO); transferrin (Tf); ultrasound (US); vascular endothelial growth factor receptor (VEGFR).

**Table 4:** Summary of successful in vivo cancer therapy using various types of PLGA NPs.

Drug@Formulation	Target (Cell Line)	Functions	Therapeutic Benefits	Ref.
As_2_O_3_@PLGA-PEG/LA NPs	Liver cancer (HepG2 cells)	EPR effect; controlled release of As_2_O_3_	1.49- and 1.09-fold reduction in tumor volume compared with saline and free As_2_O_3_, respectively, in HepG2 tumor-bearing mice	[147]
CBP/ICG@FA-PEG-PLGA NPs	Breast cancer (MCF-7 cells)	EPR effect; targeted delivery via the folate receptor; combination of chemo, photodynamic, and photothermal therapy	The strongest tumor growth suppression potentials in NPs with NIR laser irradiation group rather than the other group	[157]
CPT@RVG-PLGA NPs	Glioblastoma (GL261-Luc2 cells)	Brain-specific delivery of CPT	Prolonged tumor doubling time and increased median survival (3.15/23 days) compared with either saline (2.46/16.5 days) or blank RVG-PLGA (2.50/19 days) in mice bearing intracranial GL261-Luc2 gliomas	[142]
CP@PLGA NPs	Colon cancer (DHD/K12PROb cells)	Higher activation of caspase-3-mediated apoptosis	Comparable reduction in tumor volume with free CP in DHD/K12PROb tumor-xenografted mice	[144]
CP@mPEG-PLGA NPs	Colorectal cancer (HT 29 cells)	EPR effect; prolonged CP residence in the systemic circulation	Increased survival rate of HT 29 tumor-bearing mice compared with saline, free CP, or blank NPs	[145]
DOX@lecithin-PLGA NPs	Glioblastoma (GB 101/8 cells)	Adsorption of apolipoprotein A-1 on the surface of the NPs and subsequent improvement of endocytosis into vascular endothelial cells via lipoprotein receptors	Reduced mean tumor area (9.6 ± 10.7 mm^2^) compared with vehicle (32.1 ± 3.8 mm^2^) and free DOX (21.7 ± 13.4 mm^2^) in rats with orthotopic glioblastoma	[141]
DOX@LMWP/PLGA NPs	Breast cancer (MCF-7/ADR cells)	Targeted nuclear delivery of DOX; tumor penetration by breaking down the diffusion barriers caused by interstitial fluid pressure	Near-complete tumor growth arrest in MCF-7/ADR tumor-bearing mice compared with vehicle, free DOX, or DOX-loaded PLGA NPs	[90]
DTX@HPLGA NPs	Lung cancer (A549-Luc cells)	Enhanced colloidal stability; superior tumor selectivity *in vivo*	Improved median survival (46 days) compared with vehicle (20 days), free DTX (34 days), and blank HPLGA NPs (24 days) in orthotopic A549-Luc lung xenografts	[143]
DTX@PLGA-PDA-TPGS NPs + NIR	Drug-resistant breast cancer (MCF-7/ADR cells)	Improved photothermal conservation by PDA; inhibition of P-glycoprotein by TPGS	Approximate 10-fold reduction in tumor size and weight compared with Taxotere^®^	[159]
DTX@ANG/GS/PLGA NPs + NIR	Glioblastoma (U87-MG cells)	DTX accumulation in the tumor; heat-induced tumor cell damage	The greatest tumor inhibition rate among all groups comprising saline, 808 nm irradiation, free DTX, GS/PLGA/DTX NPs, and ANG/GS/PLGA/DTX NPs.	[130]
GEM/BA@mPEG-PLGA NPs	Ehrlich ascites carcinoma (EAC cells)	Combination drug delivery; improved pharmacokinetic properties	Reduced mean tumor volume (195.5 mm^3^) compared with saline (1236.5 mm^3^), GEM solution (553.1 mm^3^), GEM NPs (367.8 mm^3^), or GEM + BA solution (213.5 mm^3^) in mice bearing Ehrlich tumors	[146]
MM@NaHCO_3_/PLGA NPs	Breast cancer (MCF-7 cells)	EPR effect; pH-responsive degradation of NPs due to CO_2_ bubbles generated from NaHCO_3_ and subsequent rapid release of MM in lysosomes	Highest tumor growth inhibition compared with vehicle, blank NPs, or MM–loaded NPs in MCF-7 tumor-xenografted mice	[150]
MTX@PANI-LT-PLGA NPs + NIR	Breast cancer (MDA-MB-231 cells)	Targeting somatostatin receptors by LT modification; hyperthermia effect	Higher tumor suppression compared with saline, free MTX, PANI PLGA NPs, MTX/PANI PLGA NPs, or MTX/PANI LT-PLGA NPs in mice	[160]
PTX@FA-PEG-PLGA NPs	Endometrial carcinoma (HEC-1A cells)	EPR effect; targeted delivery via the folate receptor	Higher anti-tumor efficacy than free PTX and non-targeted NPs in mice	[148]
PTX@AS1411-PEG-PLGA NPs	Glioma (C6 cells)	Targeted delivery to the tumor and angiogenic blood vessels by AS1411 aptamer	The highest average tumor inhibition based on tumor volume and weight (81.68 and 79.93%), compared with non-targeted NPs (66.95 and 68.69%) and Taxol^®^ (68.69 and 46.75%)	[140]
PTX@iNGR-PEG-PLGA NPs	Glioblastoma (U87-MG cells)	Targeted delivery to angiogenic blood vessels and tumor penetration by iNGR	Prolonged median survival (42.5 days) compared with saline (17.5 days), PTX-loaded PEG-PLGA NPs (27 days), Taxol^®^ (21.5 days), and PTX-loaded cNGR-PEG-PLGA NPs (34 days) in mice bearing intracranial U87-MG glioblastoma	[149]

Adriamycin (ADR); angiopep-2 (ANG); betulinic acid (BA); camptothecin (CPT); carboplatin (CBP); cisplatin (CP); CNGRC peptide (cNGR); CRNGRGPDC peptide (iNGR); d-α-tocopheryl polyethylene glycol 1000 succinate (TPGS); docetaxel (DTX); doxorubicin (DOX); enhanced permeability and retention (EPR); folic acid (FA); fragment of rabies virus coat protein (RVG); gemcitabine (GEM); gold nanoshell (GS); hyaluronic acid-coated PLGA (HPLGA); indocyanine green (ICG); lactose acid (LA); lanreotide (LT); low molecular weight protamine (LMWP); luciferase (Luc); methenamine mandelate (MM); methotrexate (MTX); methoxypolyethylene glycol (mPEG); near-infrared (NIR); paclitaxel (PTX); polyaniline (PANI); polydopamine (PDA); polyethylene glycol (PEG).

**Table 5:** Summary of in vivo pharmacokinetic parameters altered by PLGA-based NPs.

Drug@Formulation	Animal Model	Pharmacokinetic Alterations	Ref.
DTX@PEG-PLGA NPs	Balb/C mice bearing C26 tumors	▪ Plasma CL (mL/h/kg): 407.1 in solution; 148.4 in DTX-PEG-PLGA NPs	[161]
PTX@P85/Tf-PLGA NPs	SD rats bearing C6 glioma	▪ Plasma AUC (μg·h/mL): 108.23 in solution; 362.52 in PLGA-NPs; 391.54 in P85-PLGA-NPs; 551.83 in Tf-PLGA-NPs ▪ Plasma t_1/2_ (h): 2.1 in solution; 3.96 in PLGA-NPs; 4.33 in P85-PLGA-NPs; 5.43 in Tf-PLGA-NPs	[162]
Lonidamine@PLGA-PEG-EGFR peptide NPs	Female nude mice xenografted with MDA-MB-231 tumors	● Plasma ▪ AUC (μg·h/mL): 768.8 in solution; 992.44 in PLGA-PEG NPs; 1316.01 in PLGA-PEG-EGFR NPs ▪ MRT (h): 2.36 in solution; 3.77 in PLGA-PEG NPs; 4.38 in PLGA-PEG-EGFR NPs ▪ t_1/2_ (h): 1.67 in solution; 3.92 in PLGA-PEG NPs; 4.16 in PLGA-PEG-EGFR NPs ● Tumor ▪ AUC (μg·h/mL): 4.58 in solution; 32.09 in PLGA-PEG NPs; 49.96 in PLGA-PEG-EGFR NPs ▪ MRT (h): 1.94 in solution; 4.46 in PLGA-PEG NPs; 7.25 in PLGA-PEG-EGFR NPs ▪ λ_z_ (h): 0.63 in solution; 0.26 in PLGA-PEG NPs; 0.13 in PLGA-PEG-EGFR NPs	[163]
Pt@PLGA-PEG NPs	SD rats	● Plasma ▪ AUC_0–24 h_ (μg·h/mL): 14.7 in Pt prodrug; 48.9 in PLGA-PEG NPs ▪ V_d_ (mL/kg): 210.9 in Pt prodrug; 43.2 in PLGA-PEG NPs ● Blood ▪ AUC_0–24 h_ (μg·h/mL): 3.8 in Pt prodrug; 12.7 in PLGA-PEG NPs ▪ V_d_ (mL/kg): 678.7 in Pt prodrug; 81.9 in PLGA-PEG NPs	[164]
ICG@PEG/FA-PLGA NPs	Female NCr mice xenografted with MDA-MB-231 tumors	▪ AUC_0–12 h_ ratio (PEG/FA-PLGA NPs: PLGA NPs): 3.45 in plasma;2.94 in tumor; 0.87 in liver	[166]
Bufalin@PEG-PLGA-PLL-RGD NPs	Mice bearing colon cancer	▪ t_1/2_ (h): 3.35 in solution; 7.17 in PEG-PLGA-PLL-RGD NPs ▪ MRT (h): 3.45 in solution; 7.63 in PEG-PLGA-PLL-RGD NPs ▪ V_d_ (L/kg): 63.37 in solution; 187.83 in PEG-PLGA-PLL-RGD NPs	[167]
DTX@PLGA/HA NPs	SD rats	▪ AUC (μg·h/L): 6110 in solution; 9394 in PLGA NPs; 23,175 in PLGA/HA NPs ▪ V_d_ (L/kg): 6.94 in solution; 7.52 in PLGA NPs; 3.16 in PLGA/HA NPs	[168]
DTX@PLGA-PEG-Apt NPs	SD rats	▪ AUC (ng·h/mL): 1393.6 in solution; 3996.9 in PLGA-PEG NPS; 3807.4 in PLGA-PEG-Apt NPs ▪ CL (L/h): 3.588 in solution; 1.251 in PLGA-PEG NPS; 1.313 in PLGA-PEG-Apt NPs ▪ V_d_ (L): 0.501 in solution; 0.165 in PLGA-PEG NPS; 0.166 in PLGA-PEG-Apt NPs	[169]

Aptamer (Apt); Arg-Gly-Asp (RGD); docetaxel (DTX); epidermal growth factor receptor (EGFR); folic acid (FA); hyaluronic acid (HA); indocyanine green (ICG); platinum (Pt); Pluronic^®^ P85 (P85); polyethylene glycol (PEG); poly-l-lysine (PLL); Sprague-Dawley (SD); transferrin (Tf).
